# Insights from the Structure of an Active Form of *Bacillus thuringiensis* Cry5B

**DOI:** 10.3390/toxins14120823

**Published:** 2022-11-23

**Authors:** Jiaxin Li, Lin Wang, Masayo Kotaka, Marianne M. Lee, Michael K. Chan

**Affiliations:** 1 School of Life Sciences and Center of Novel Biomaterials, The Chinese University of Hong Kong, Shatin, Hong Kong SAR 999077, China; 2 School of Biomedical Sciences, LKS Faculty of Medicine, The University of Hong Kong, Pokfulam, Hong Kong SAR 999077, China

**Keywords:** Cry5B, crystal structure, nematicidal activity, oligomerization

## Abstract

The crystal protein Cry5B, a pore-forming protein produced by the soil bacterium *Bacillus thuringiensis*, has been demonstrated to have excellent anthelmintic activity. While a previous structure of the three-domain core region of Cry5B(112–698) had been reported, this structure lacked a key N-terminal extension critical to function. Here we report the structure of Cry5B(27–698) containing this N-terminal extension. This new structure adopts a distinct quaternary structure compared to the previous Cry5B(112–698) structure, and also exhibits a change in the conformation of residues 112–140 involved in linking the N-terminal extension to the three-domain core by forming a random coil and an extended α-helix. A role for the N-terminal extension is suggested based on a computational model of the tetramer with the conformation of residues 112–140 in its alternate α-helix conformation. Finally, based on the Cry5B(27–698) structure, site-directed mutagenesis studies were performed on Tyr495, which revealed that having an aromatic group or bulky group at this residue 495 is important for Cry5B toxicity.

## 1. Introduction

Crystal (Cry) proteins produced during sporulation by the spore-forming soil bacterium *Bacillus thuringiensis* (*Bt*) are best known for their ability to serve as natural insecticides. The specificity of Cry proteins to insects, and the absence of their toxicity towards mammals and other vertebrates have contributed to the incorporation of their genes into various transgenic crops for protection against insect infestations [1,2,3]. In recent years, a select group of Cry proteins has been identified and shown to be toxic to nematodes. One of the most extensively characterized of these is Cry5B, which has been demonstrated to be highly active in vitro and in vivo against multiple nematodes including free-living and parasitic species [4]. Plant-parasitic nematodes, such as root-knot nematodes, cyst nematodes and lesion nematodes, annually cause a hundred billion US dollars in damage to crops worldwide, and thus there is interest in combining the use of Cry5B together with other insecticidal Cry proteins in order to protect the agricultural crop production to provide broader protection against crop pests.

Parasitic nematode infections also occur in humans and animals, and thus there has been interest in exploring their use as a therapy to treat intestinal nematode infections with some promising results having been obtained in early in vivo studies [5,6,7,8]. Oral administration of purified Cry5B crystals to *Ancylostoma ceylanicum*-infected hamsters elicited a comparable effect to the standard anthelminthic drug mebendazole [5], while *Bt* lysate with cytosolic Cry5B treatment of pigs infected with *Ascaris suum* showed 97% elimination of L4 larvae [6]. These findings support the use of Cry5B as a potential drug for treating livestock and human nematode infections.

The nematicidal activity of Cry5B has been proposed to follow a sequence of events similar to those of the insecticidal Cry toxins binding to a host receptor, followed by membrane insertion that causes intestinal damage and ultimately the death of the host [9]. Previous investigations on the toxicity of Cry5B against *Caenorhabditis elegans* have shown that Cry5B binds to a glycolipid comprised of an invertebrate-specific tetrasaccharide core *N*-acetylgalactosamine (GalNAc) β1–4 *N*-acetylglucosamine (GlcNAc) β1–3 mannose (Man) β1–4 glucose (Glc) attached to the lipids on the surface of *C. elegans’* intestinal epithelial cells [10,11]. How the binding affects the Cry5B conformation and/or triggers the oligomerization is currently unclear.

A structure of an elastase-treated Cry5B encompassing residues 112–698 has been reported [11]. The structure revealed that Cry5B(112–698) possesses a three-domain core similar to that found in the structures of other Cry proteins (e.g., Cry1Ac, Cry3Aa, Cry4Ba). In these structures, domain I consists of a helix bundle, domain II adopts a β-prism motif, and domain III has a β-sandwich motif. Notably, the fold of domain II in Cry5B exhibits similarity to banana lectin (BanLec), a mannose-specific jacalin-related lectin whose two separate glycan-binding sites are located in the loop regions on the top of the β-prism [12]. The glycan-binding sites in BanLec are made up of loops with a GG or GXXXD motif, while in Cry5B domain II they are conserved as GG and GXXXE, respectively.

Given that proper proteolytic activation is required to convert Cry protoxin to its toxic counterpart, and the fact that previous studies found that while C-terminal truncation of Cry5B at residue 698 does not affect its toxicity to *C. elegans*, truncation of the N-terminus before nucleotide 63 (residue 21) weakens the toxicity dramatically [13], a key question with respect to the Cry5B(112–698) protein was whether this elastase-treated Cry5B construct retained its toxicity to *C. elegans*. We subsequently confirmed that Cry5B(1–698) was active as reported [13] and exhibited similar nematicidal activity as Cry5B(1–772), while Cry5B(112–698) was inactive (Figure S1). The significance of these findings was the implication that the N-terminus of Cry5B contains a feature critical to function. We thus sought to determine the structure of Cry5B containing its N-terminal region and herein report such a structure of Cry5B(27–698). In addition to the N-terminal extension, the newly determined crystal structure reveals a network of inter-residue hydrogen bonding mediated by Tyr495; we thus evaluated the functional significance of Tyr495 by site-directed mutagenesis and showed that mutation of Tyr495 can significantly impact the toxicity of Cry5B against *C. elegans*.

## 2. Results

### 2.1. Structure of Cry5B(27–698) with Its Functionally Required N-Terminal Extension

Recombinant Cry5B(1–772) with an N-terminal His-tag was overexpressed in *E. coli* and the soluble protein was purified by Ni-affinity and gel-filtration chromatography (Figure S2). Screening of crystallization conditions led to the identification of protein crystals that diffracted to 4.5 Å resolution. The structure was determined by molecular replacement using the Cry5B(112–698) (PDB ID: 4D8M) structure as the initial model and found to contain two molecules in the asymmetric unit (Table 1).

**Figure 1:**
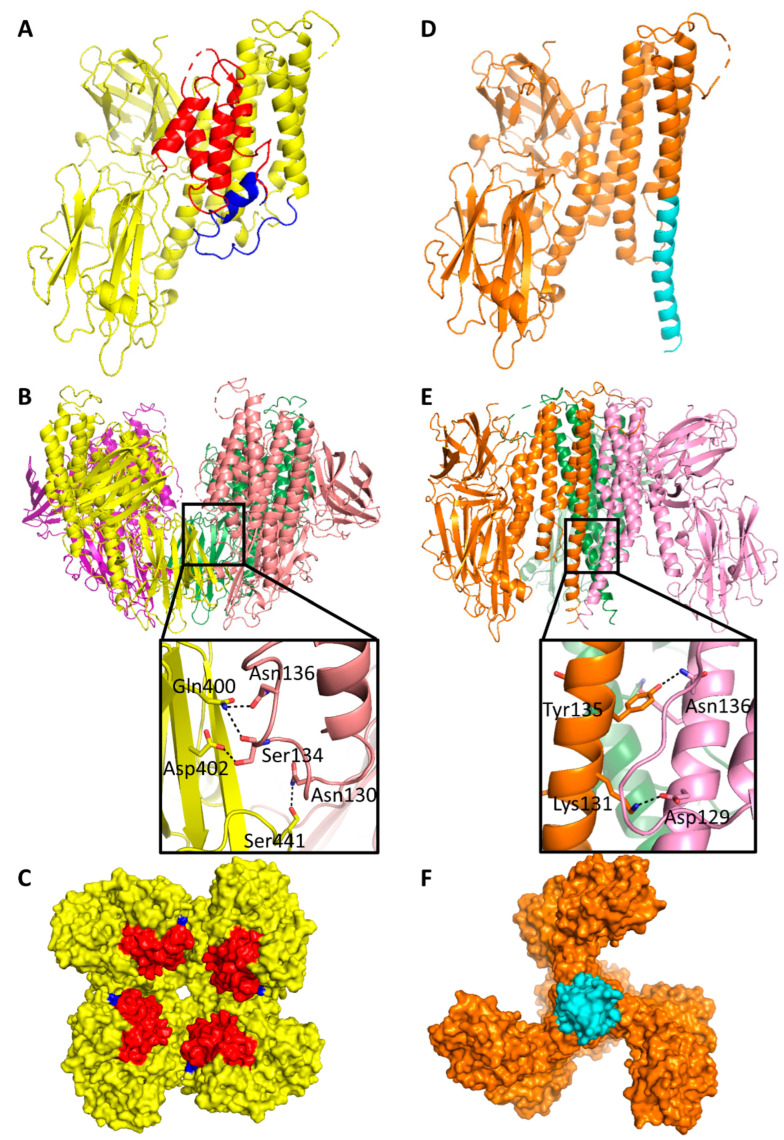
Structures of Cry5B(27–698) and Cry5B(112–698). Structure of (**A**) single subunit and tetramer in (**B**) side view and (**C**) top view of Cry5B(27–698). N-terminal functional extension (residues 27–107) in red, residues 112–140 in blue and the three-domain core (residues 141–698) colored in yellow. Structure of (**D**) single subunit and trimer in (**E**) side view and (**F**) bottom view of Cry5B(112–698) (PDB ID: 4D8M). Residues 112–140 in cyan and the three-domain core (residues 141–698) colored in orange. The black boxes in (**B**,**E**) showing the interaction between neighboring subunits. Hydrogen bond shown as black dashed lines.

The dominant feature of the new Cry5B(27–698) structure is the three-domain core (residues 141–698) observed in the previous elastase-treated Cry5B(112–698) structure. A new region does not present in previously determined Cry5B structures; however, it is an N-terminal extension attached to the three-domain core consisting of five short helices buttressed against a pocket formed by domains I and II. While an initial trace could be generated, the assignment of residues based on the electron density map was hindered by the low resolution. Thus to assist in the residue assignments of this N-terminal extension, the amino acid sequence of residues 1–134 was submitted to Robetta [14] to generate a predicted model. Notably, the resulting Robetta model fitted the polyalanine model built based on the electron density quite well for at least the first four helices. Using this Robetta model to guide the side chain assignments, a final model was obtained spanning residues 27–698 (Figure 1A). N-terminal additional helices were assigned as α1(29–39), α2(50–61), α3(71–78), α4(91–97) and α5(115–122). Residues 1–24 were predicted by Robetta to be flexible loops with two very short helices, and thus we presume residues 1–26, which could not be modeled, are disordered. No density for amino acids beyond residue 698 was observed, suggesting either their disorder or cleavage during crystallization. The presence of aromatic amino acids (Phe, Trp and Tyr) with strong electron density, and the location of Pro and Gly residues at the turns were also used to guide the sequence alignment and side chains were fitted to the electron density map to the best extent possible. The positions of residues 141–698 were based on the existing Cry5B(112–698) structure used for molecular replacement.

In addition to the presence of the N-terminal extension, another significant difference between our Cry5B(27–698) and the elastase-treated Cry5B(112–698) structures is in the orientation and secondary structure conformations of residues 112–140. In the previous Cry5B(112–698) structure, these residues form part of an extended α-helix (residues 112–162) that helps to tighten the homotrimer (Figure 1D,E). However, in our new structure, residues 113–127 form an α-helix that lies adjacent to the short helices of the N-terminal extension, while residues 128–140 unwind to adopt a random coil (Figure 1A). Presumably this unwinding is needed to accommodate the N-terminal extension and remain connected to the three-domain core.

**Figure 2:**
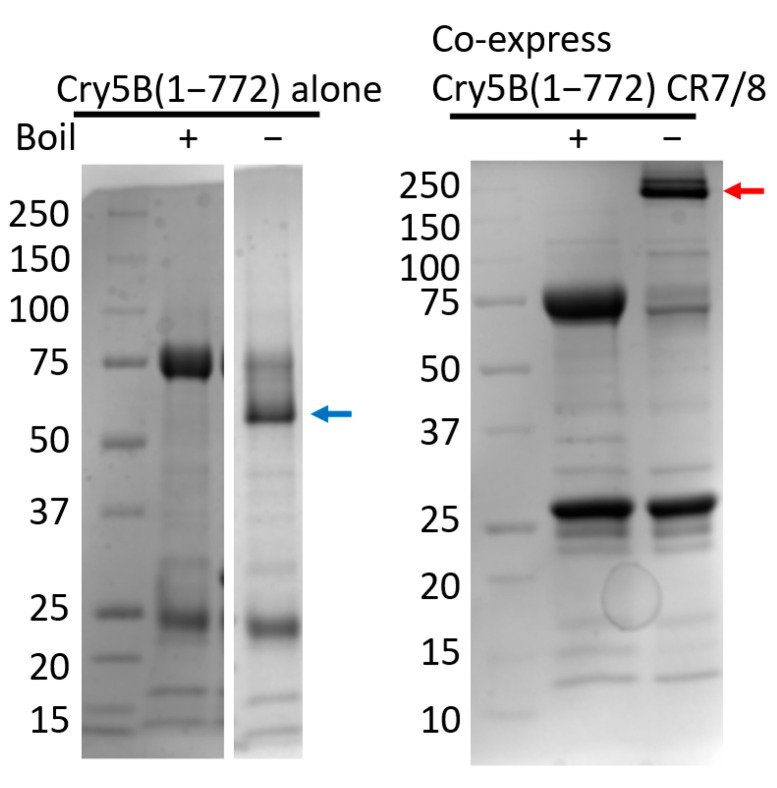
CDH-8 CR7/8 can trigger the in vitro oligomerization of Cry5B(1–772). The blue and red arrows indicate the Cry5B(1–772) monomer and oligomer in native condition, respectively.

Arguably the biggest difference in the two Cry5B structures, however, is the oligomerization state of the protein. While the elastase-treated Cry5B(112–698) structure forms a trimer with no apparent channel (Figure 1E,F), which is similar to that observed for Cry4Ba [15,16,17], our Cry5B(27–698) structure appears to form a weakly associated tetramer with a wide opening that narrows into a 10 Å pore (Figure 1B,C). Considering the interaction of Cry5B with cadherin sequences has been shown to lead to a tetrameric species [18], we also verified that Cry5B(1–772) can oligomerize when co-expressing with cadherin repeats 7 and 8 (CD7/8) from nematode-specific cadherin CDH-8 (Figure 2). It is possible that the cadherin peptides can strengthen the interaction between Cry5B subunits and stabilize a tetramer similar to that observed in the structure.

### 2.2. Cry5B (27–698) Is an Active Toxin

**Figure 3:**
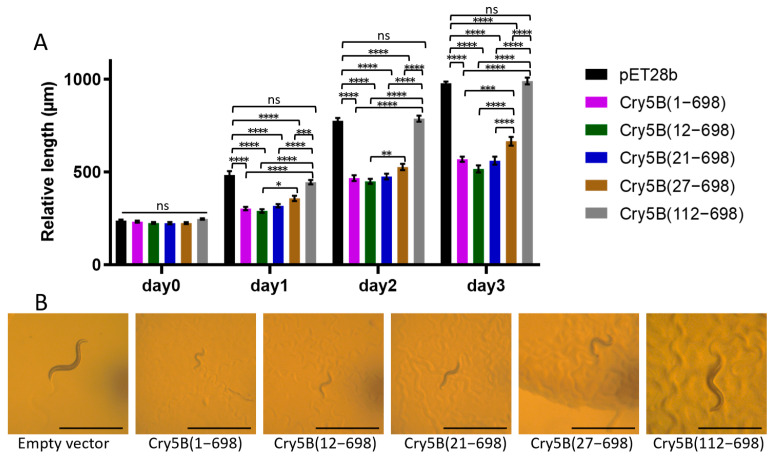
Toxicity test of Cry5B N-terminal truncations against *C. elegans*. (**A**) Relative length of worms indicated the toxicity of Cry5B constructs. (**B**) Each panel shows a typical *C. elegans* grown on *E. coli* BL21 expressing different constructs after three days. Scale bar represents 1 mm. Worms grown on empty vector and Cry5B(112–698) are obviously large and healthy, while those grown on Cry5B(1–698), Cry5B(12–698), Cry5B(21–698) and Cry5B(27–698) are much smaller, indicating intoxication. Two-way ANOVA, N = 15–25, **** *p* < 0.0001, *** *p* < 0.001, ** *p* < 0.01, * *p* < 0.05; ns, not significant.

As mentioned above, the previous structure of Cry5B(112–698) was of an inactive form of the toxin. Based on the structure, the shortest N-terminal truncation of the protein forming the crystal used for the Cry5B(27–698) would be at residue 26, though additional residues could be present but not observed due to disorder. To determine whether Cry5B(27–698) represents an active form, *E. coli* expressing Cry5B with different truncations, Cry5B(1–698), Cry5B(12–698), Cry5B(21–698), Cry5B(27–698) and Cry5B(112–698), were fed *C. elegans* to see which were toxic (Figure 3 and Figure S3A). These results confirmed that Cry5B(112–698) is non-toxic, while N-terminal truncations before residue 21 showed no effect. Importantly, while the Cry5B(27–698) construct showed a statistically significant reduction in the toxicity compared to constructs with the shorter N-terminal truncation, it still remained highly active and showed much higher toxicity than Cry5B(112–698). These data indicate that the Cry5B(27–698) structure represents an active form of the toxin.

### 2.3. Identification of Tyr495 as a Residue with a Key Role in the Nematicidal Activity of Cry5B

**Figure 4:**
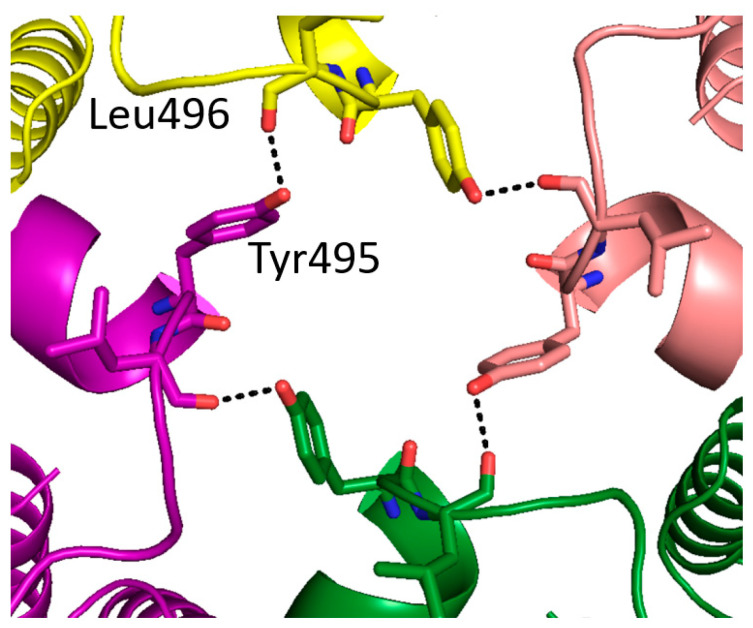
A ring of hydrogen bonds at the apex of the tetramer Cry5B(27–698). Four subunits colored as in Figure 1B. Hydrogen bonds shown as black dashed lines.

**Figure 5:**
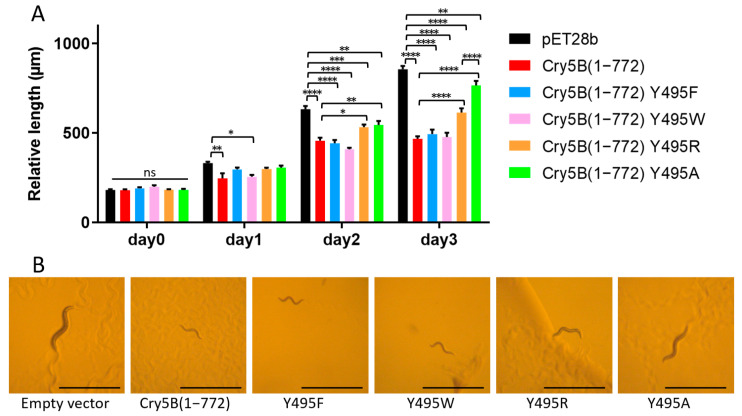
Toxicity test of Cry5B(1–772) Y495 mutants against *C. elegans*. (**A**) Relative length of worms indicated the toxicity of Cry5B constructs. (**B**) Each panel shows a typical *C. elegans* grown on *E. coli* BL21 expressing different constructs after three days. Scale bar represents 1 mm. Worms grown on empty vector and Cry5B(1–772) Y495A are obviously larger, while those grown on wild-type Cry5B(1–772), Cry5B(1–772) Y495F, Cry5B(1–772) Y495W and Cry5B(1–772) Y495R are much smaller. Two-way ANOVA, N = 10, **** *p* < 0.0001, *** *p* < 0.001, ** *p* < 0.01, * *p* < 0.05; ns, not significant.

In the course of analyzing the putative tetramer of our Cry5B structure, we noticed that Tyr495 formed a hydrogen bond to a neighboring Leu carbonyl, which together with the 4-fold symmetry, formed a ring of hydrogen bonds at the apex of the tetramer (Figure 4). We thus decided to evaluate whether this residue and the relevant inter-residue interactions were important. Tyr495 was therefore substituted with Ala and the toxicity of the corresponding protein was investigated (Figure 5 and Figure S3B). It was found that a mutation of Tyr495 to Ala in the Cry5B(1–772) construct resulted in the loss of nematicidal activity, demonstrating the importance of this residue. Previous studies of Cry1Ab identified a Phe residue involved in membrane insertion, which lost activity when mutated to Ala but retained activity when mutated to other aromatic residues [19,20]. We thus prepared Phe and Trp mutants of Tyr495 and found that these mutations resulted in retention in activity as well (Figure 5). Replacing Tyr495 with an Arg appeared to retain some activity, though there was a statistically significant decrease compared to the wild type (Figure 5). Collectively, these data suggested that the hydrogen bond in this region was not critical for activity, and instead that having an amino acid with an aromatic or possibly bulky group at residue 495 is important for function.

## 3. Discussion

### 3.1. Computational Model for the N-Terminal Extension in Cry5B Tetramer

As mentioned previously, one surprising difference in our Cry5B(27–698) structure and the previous elastase-treated Cry5B(112–698) structure is the location and conformation of residues 112–140 (Figure 1A,D). In the Cry5B(112–698) structure, this region forms an extended helix (residues 112–162) belonging to the five-helix bundle. In our Cry5B(27–698) structure, however, it appears to be partially unwound, connecting the N-terminal extension (residues 27–107), which was not present in Cry5B(112–698), to domain I of the three-domain core.

**Figure 6:**
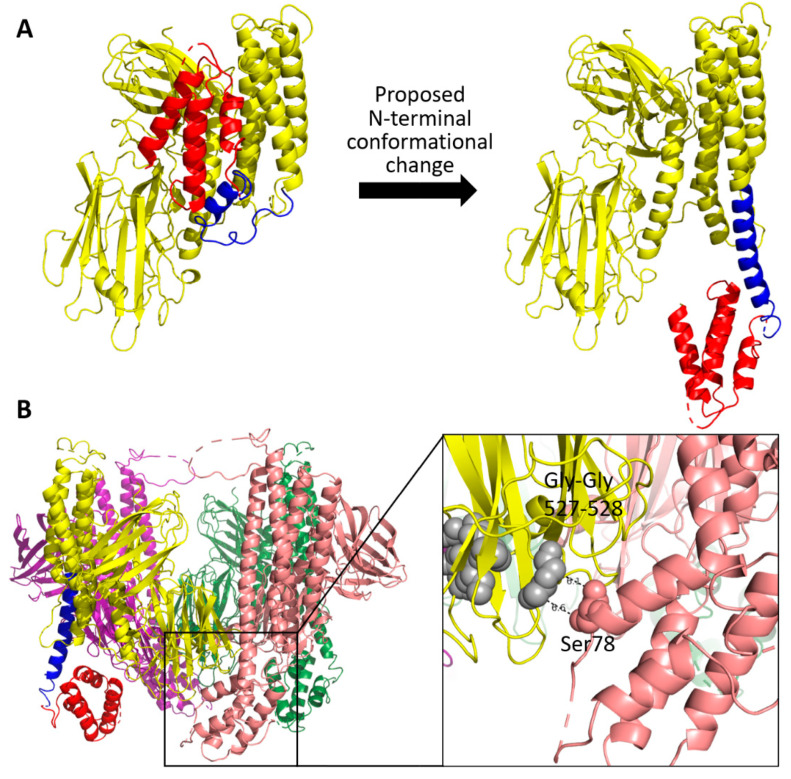
Computational model of the Cry5B(27–698) with extended helix. (**A**) Proposed reorientation of N-terminal region. (**B**) Modeled Cry5B(27–698) tetramer structure when N-terminal region rearranged to form an extended helix (residues 112–162). Colored as in Figure 1A. Black boxes showing the close distance (~7 Å) between the proposed glycan-binding motif (GG and GPIEE shown as grey spheres) and the possible position of N-terminal region (red).

Given that forming the extended α-helix(112–140) observed in the Cry5B(112–698) should be enthalpically favorable, we decided to produce a computational model of the tetrameric structure of Cry5B(27–698) with an extended helix (residues 112–162) to see where the N-terminal extension (residue 27–107) would be located. The model shown in Figure 6 reveals that in the tetramer, the rearranged N-terminal region would be positioned near (~7 Å) the proposed glycan-binding motif in domain II [11] of a neighboring subunit. Given that the N-terminal extension is important for eliciting the nematicidal activity of Cry5B, we wondered whether the possible rearrangement could provide a mechanism for the N-terminal extension to impact glycan binding, and decided to test this hypothesis by further mutagenesis and functional analyses.

### 3.2. Cry5B N-Terminal Region (Residues 27–111) Contributes to Binding with Galactose

Considering the galactose-dependent binding mechanism between Cry5B and glycolipid in *C. elegans*, and the fact that galactose can inhibit their binding [10], the binding affinity between the Cry5B constructs (residues 27–698 and 112–698) with galactose was determined by microscale thermophoresis (MST). The data show that the dissociation constant (Kd) between Cry5B(27–698) and galactose was 1.64 ± 1.04 μM, while that for Cry5B(112–698) was 21.5 ± 10.3 μM, suggesting a 10-fold weaker binding (Figure S4). These data support the notion that the N-terminal region (residues 27–111) of Cry5B is likely involved in its binding with the glycolipid receptor with one possibility being the rearrangement of the N-terminal extension predicted by the computational model. Further insights could be obtained from the complex structure of Cry5B(27–698) and galactose.

### 3.3. Comparison of the Cry5B(27–698) with Other Three-Domain Cry Proteins

The toxicity studies of different N-terminal truncations of Cry5B (Figure 3) showed that N-terminal truncations before residue 21 had no impact on its toxicity, and that Cry5B(27–698) was still active though less toxic, while Cry5B(112–698), which lacks the N-terminal extension, showed complete loss of activity, thus highlighting the importance of the N-terminal extension on the function of Cry5B. Notably, similar N-terminal helical extensions are present in other Cry proteins. For Cry1A proteins, a widely used toxin against lepidopteran insects, the presence of these N-terminal regions was also shown to be functionally important. For Cry1Ai, a construct consisting of residues (36–605) (corresponding to residues 36–606 in Cry1Ac) was found to be its minimal active fragment. Loss of one N- or C-terminal residue of this construct abolished its toxicity against *P. xylostella* larvae [21]. In another Cry1A protein, Cry1Ah(50–639) was toxic, but losing residues 50–108 abrogated its toxicity against *P. xylostella* larvae [22]. Significantly, sequence alignment of these Cry1A proteins to the structurally determined Cry1Ac proteins (Figure S5) shows the presence of an N-terminal extension whose truncation suggests a similar requirement for activity. Our findings with the Cry5B(27–698) structure corroborate these previous results and reaffirm the vital role the N-terminal regions that extend from the Domain I of three-domain Cry proteins play in the activity of Cry proteins against both insects and nematodes. Notably, many other structures of Cry proteins have been shown to contain a similar N-terminal extension (Figure S6), which could be similarly important for their function.

## 4. Conclusions

In summary, we determined the structure of an active form of Cry5B that contains an N-terminal extension encompassing residues 27–107 that is absent in the previously determined Cry5B structures. The Cry5B(27–698) structure allowed us to elucidate the structural features of this N-terminal extension, and suggests a possible role in eliciting its toxicity toward nematodes. This structure led to the identification of Tyr495 as a residue critical for Cry5B nematicidal function. These studies could help to improve the use of Cry5B as a treatment for gastrointestinal nematode infections and for preventing nematode damage on crops.

## 5. Materials and Methods

### 5.1. Cloning of Cry5B Fragments and Mutants

Cry5B(1–772) gene was amplified from *Bt* strain YBT-1518 genomic DNA by PCR using KAPA HiFi polymerase using primers Cry5B 1-EcoRI-F and Cry5B 772-XhoI-R in Table S1. The PCR products were then ligated into the EcoRI and XhoI sites of the double digested pET28b vector using Gibson Assembly. Other fragments of Cry5B such as Cry5B(1–698), Cry5B(12–698), Cry5B(21–698), Cry5B(27–698) and Cry5B(112–698) were cloned using similar strategy with corresponding primers listed in Table S1.

Using the wild-type pET28b-Cry5B(1–772) plasmid as a template, different mutations at Tyr495 were introduced using site-directed mutagenesis. Primers for each construct are listed in Table S1.

### 5.2. Expression and Purification of Cry5B(1–772)

A pET28b plasmid containing the *cry5B(1–772)* gene was transformed into *E. coli* BL21(DE3) and the recombinant protein was overexpressed in Luria–Bertani (LB) media supplemented with 50 μg/mL kanamycin and induced with 0.1 mM isopropyl β-D-1-thiogalactopyranoside (IPTG) at 20 °C and grown overnight. The harvested cells were lysed by sonication in buffer A (20 mM Tris pH 8.0, 500 mM NaCl, 0.1% phenylmethylsulfonyl fluoride (PMSF) and 0.1% benzydamine) and loaded on to a 5 mL Ni-NTA column (GE Healthcare), which was then washed with buffer containing a 0 to 500 mM imidazole gradient. The recombinant Cry5B(1–772) protein was eluted at ~150 mM imidazole and further purified by size-exclusion chromatography (GE Healthcare) with buffer B (20 mM HEPES pH 8.0, 50 mM NaCl). The fractions were analyzed by SDS-PAGE and checked by mass spectrometry-based protein identification.

### 5.3. Crystallization and Data Collection

Cry5B(1–772) was crystallized using the sitting-drop vapor-diffusion method from plates initially incubated at 18 °C, and then transferred to 32 °C after 2 weeks. Several crystals were obtained from similar conditions of the Cubic Screen (Hampton) containing 2500 mM NaCl as precipitant. The crystal used for data collection was obtained from condition with 2500 mM NaCl, 100 mM sodium cacodylate pH 6.5 and 200 mM Li_2_SO_4_. The diffraction dataset was collected on beamline 13B at the NSRRC in Hsinchu, Taiwan. Data processing and scaling were performed with iMosflm [23] and SCALA [24] from the CCP4i suite [25].

### 5.4. Phase Determination and Refinement

The initial structure was generated using PHASER [26] using the structure of Cry5B(112–698) (PDB ID: 4D8M) as searching model. The extra N-terminal region beyond this model was manually added and adjusted to fit the electron density using the program Coot [27]. To assist in the side chain identification, the N-terminal sequence (residues 1–134) was submitted to Robetta [12], which was used to generate a predicted model. In addition to guiding the side chain assignments, some density at the ends of helices that we had initially fitted as random coils were recognized as being helical when Robetta model was used for comparison. Iterative cycles of model building and refinement with the programs Coot and Phenix [28], respectively, were carried out to improve the model. The quality of the final model was evaluated using the program MolProbity [29] and summarized in Table 1. The coordinates and structure factors have been deposited in the Protein Data Bank (PDB ID: 8HHE).

### 5.5. Cry5B Toxicity Study on C. elegans

#### 5.5.1. General Preparation

The *C. elegans* strain Bristol N2 and *E. coli* OP50 used in this study were a gift from Professor King-Lau Chow of HKUST. Nematode growth media (NGM) was prepared according to published protocol [30], and the *C. elegans* worms were grown on NGM plates inoculated with 100 μL *E. coli* OP50 overnight culture. After 3–5 days’ growth and reproduction at 20 °C, the worms were synchronized based on the protocol [30], and the larvae harvested at the L1–L2 stage were used in toxicity test.

#### 5.5.2. Toxicity Test

*E. coli* BL21 transformed with pET28b vectors harboring either Cry5B(1–772) or one of its Y495 mutants or Cry5B(1–698) or one of its N-terminal truncations were induced with 0.1 mM IPTG and grown at 20 °C in 5 mL LB culture. The overnight culture supplemented with an additional 5 mM IPTG was used to inoculate NGM plates. The plates seeded with *E. coli* were incubated at 20 °C overnight before use. For the control group, empty pET28b vector was used.

Five synchronized L1–L2 larvae were transferred to each NGM plate. The appearance of worms was monitored for three days. As the toxin inhibits the growth of worms, the relative length of *C. elegans* reflects the toxicity of the Cry5B constructs. Photographs of every worm were taken every 24 h. The length was determined using the software ImageJ [31], and data were analyzed using software GraphPad Prism 6.

#### 5.5.3. Microscale Thermophoresis (MST)

Purified His-Cry5B(27–698) and His-Cry5B(112–698) as protein targets were labelled using Alexa Fluor 647 NHS ester dye according to the instruction. Unreacted dye was removed with a desalting column (30K MWCO, Bio-Rad). The labelled targets were adjusted to appropriate concentrations for detection, and the galactose as ligand was freshly solubilized in the same buffer—aforementioned buffer B (20 mM HEPES pH 8.0, 50 mM NaCl).

For each assay, 16 different serially-diluted concentrations of ligand were firstly prepared, and then mixed with equal volume of labelled protein target at room temperature. The reaction mixtures were loaded into standard Monolith NT.115 capillaries and measured using a Monolith NT.115 instrument (NanoTemper Technologies). Instrument parameters were adjusted to 40% MST power and 2% or 6% excitation power (2% for Cry5B(27–698) and 6% for Cry5B(112–698). The Kd values were calculated using MO.Affinity Analysis v.2.2.4 software (NanoTemper Technologies) as mean ± SEM from at least three independent experiments with a single site-specific binding model.

## Figures and Tables

**Table 1:** Data Collection and Refinement Statistic.

	Cry5B(27–698)
**Data collection**	
Wavelength (Å)	0.99984
Space group	P 4 21 2
Cell dimensions: *a*, *b*, *c* (Å)	114.4 114.4 263.4
α, β, γ (°)	90.0 90.0 90.0
Resolution (Å)	20 (4.5)
*R*_merge_	0.274 (0.559)
*I*/σ*I*	2.2 (1.3)
Completeness (%)	99.7 (100)
Redundancy	5.3 (5.4)
**Refinement**	
No. reflections	10,836
*R*_work_/*R*_free_	23.76/28.85
No. atoms: Protein	10,254
Ligand/ion	0
Water	0
*B*-factors(Å^2^): Protein	107.08
R.m.s. deviations: Bond lengths (Å)	0.002
Bond angles (°)	0.54
Range of residues	27–83, 88–107, 113–165, 173–214, 224–698

Outer shell statistics are in parentheses.

## Data Availability

The atomic coordinates and structure factors of Cry5B(27–698) have been deposited in the protein data bank under the accession codes 8HHE.

## Supplementary Materials

The following supporting information can be downloaded at: https://www.mdpi.com/article/10.3390/toxins14120823/s1, Figure S1: Toxicity test of Cry5B(1–698), Cry5B(1–772) and Cry5B(112–698) against *C. elegans*. Figure S2: Purified Cry5B(1–772). Figure S3: Western blot detects His-tag indicating the expression level of His-Cry5B constructs. Figure S4: Binding curves from MST assays for the binding of Cry5B constructs and galactose. Figure S5: Sequence alignment of Cry1A proteins. Figure S6: Structures of three-domain Cry proteins possessed with the extra amino acid beyond five-helix bundle. Table S1: Primers used for making Cry5B constructs.
