# Supplementary material for: Insights from the Structure of an Active Form of Bacillus thuringiensis Cry5B
Source: Toxins (Basel). 2022 Nov 23;14(12):823. doi: 10.3390/toxins14120823 (PMC9785347; doi:10.3390/toxins14120823)
Supplement: Supplementary file 1 [file toxins-14-00823-s001.zip › toxins-2034515-supplementary.pdf]

toxins MDPI

Supplementary Materials

Insights from the Structure of an Active Form of *Bacillus thuringiensis* Cry5B

Jiaxin Li [1,†], Lin Wang [1,†], Masayo Kotaka [1,2], Marianne M. Lee [1] and Michael K. Chan [1,*].

1 School of Life Sciences and Center of Novel Biomaterials, The Chinese University of Hong Kong, Shatin, Hong Kong SAR 999077, China
2 School of Biomedical Sciences, LKS Faculty of Medicine, The University of Hong Kong, Pokfulam, Hong Kong SAR 999077, China
* Correspondence: michaelkchan88@cuhk.edu.hk; Tel.: +852-39431487
† These authors contributed equally to this work.

This document includes:

Figure S1. Toxicity test of Cry5B(1–698), Cry5B(1–772) and Cry5B(112–698) against *C. elegans*.
Figure S2. Purified Cry5B(1–772).
Figure S3. Expression of His-Cry5B constructs in *E. coli* fed to *C. elegans*.
Figure S4. Binding curves from MST assays for the binding of Cry5B constructs and galactose.
Figure S5. Sequence alignment of Cry1A proteins.
Figure S6. Structures of three-domain Cry proteins possess with the extra amino acid beyond five-helix bundle.
Table S1. Primers used for making Cry5B constructs.

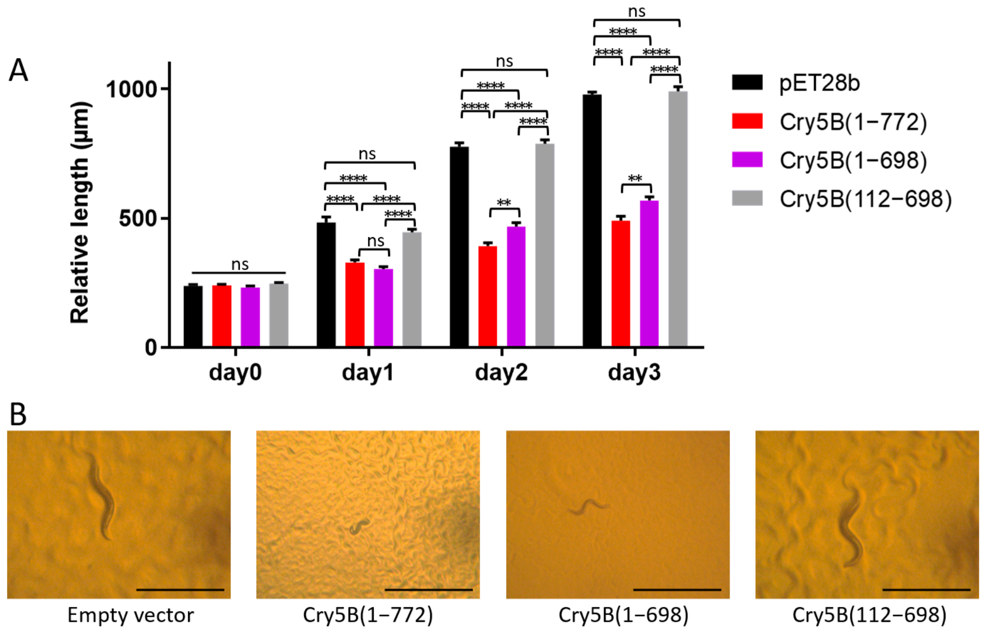

Figure S1. Toxicity test of Cry5B(1–698), Cry5B(1–772) and Cry5B(112–698) against *C. elegans*. (**A**) Toxicity of Cry5B truncations on *C. elegans* reflected by the relative length of worms. (**B**) Each panel shown a typical *C. elegans* grown on *E. coli* BL21 expressing different constructs after three days. Scale bar represents 1 mm. Worms grown on empty vector and Cry5B(112–698) are relatively large and healthy while those grown on Cry5B(1–772) and Cry5B(1–698) are much smaller, indicating intoxication. Two-way ANOVA, N=15–25, **** P < 0.0001, ** P < 0.01, ns non-significant.

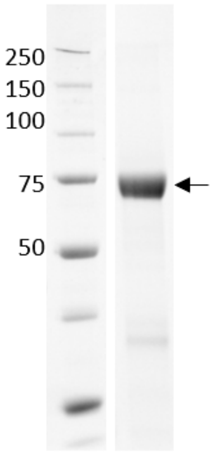

Figure S2. Purified Cry5B(1–772). Arrow indicates target protein.

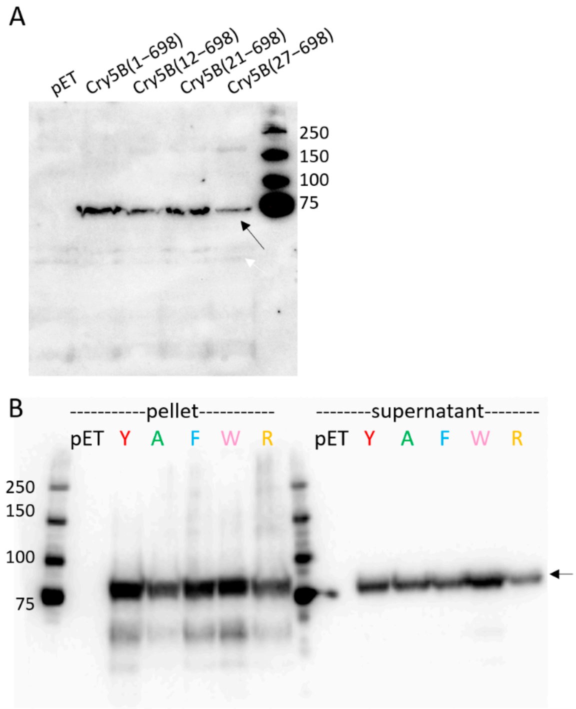

Figure S3. Expression of His-Cry5B constructs in *E. coli* fed to *C. elegans*. The presence of each Cry5B construct was verified by detection of its N-terminal His-tag by Western blot. (**A**) Cry5B N-terminal truncations. (**B**) Cry(1–772)Y495 mutants with the lane labeled with the single letter code of the amino acid at residue 495. The arrow indicates the bands corresponding to His-Cry5B.

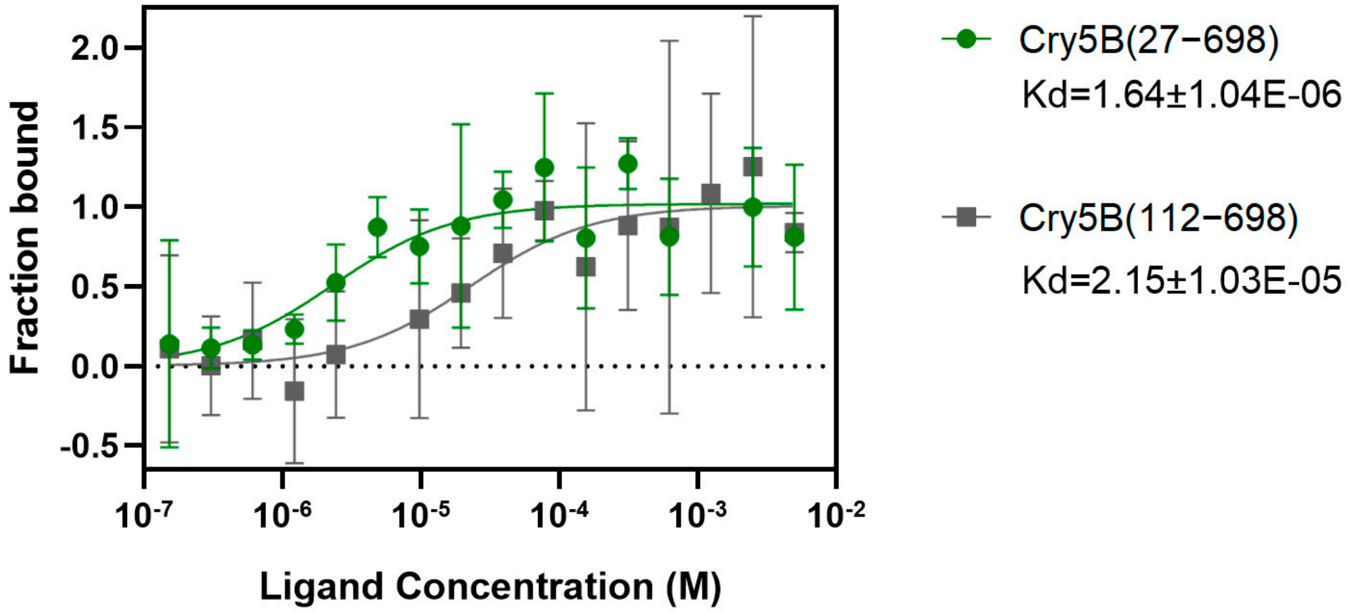

Figure S4. Binding curves from MST assays for the binding of Cry5B constructs and galactose. Figure is plotted using GraphPad Prism 8 software, with error bars represent the SEM of one independent experiment with at least three technical repeats, three for Cry5B(27–698) and four for Cry5B(112–698). Kd calculated using MO.Affinity Analysis v.2.3 software (NanoTemper Technologies) with a single site-specific binding model.

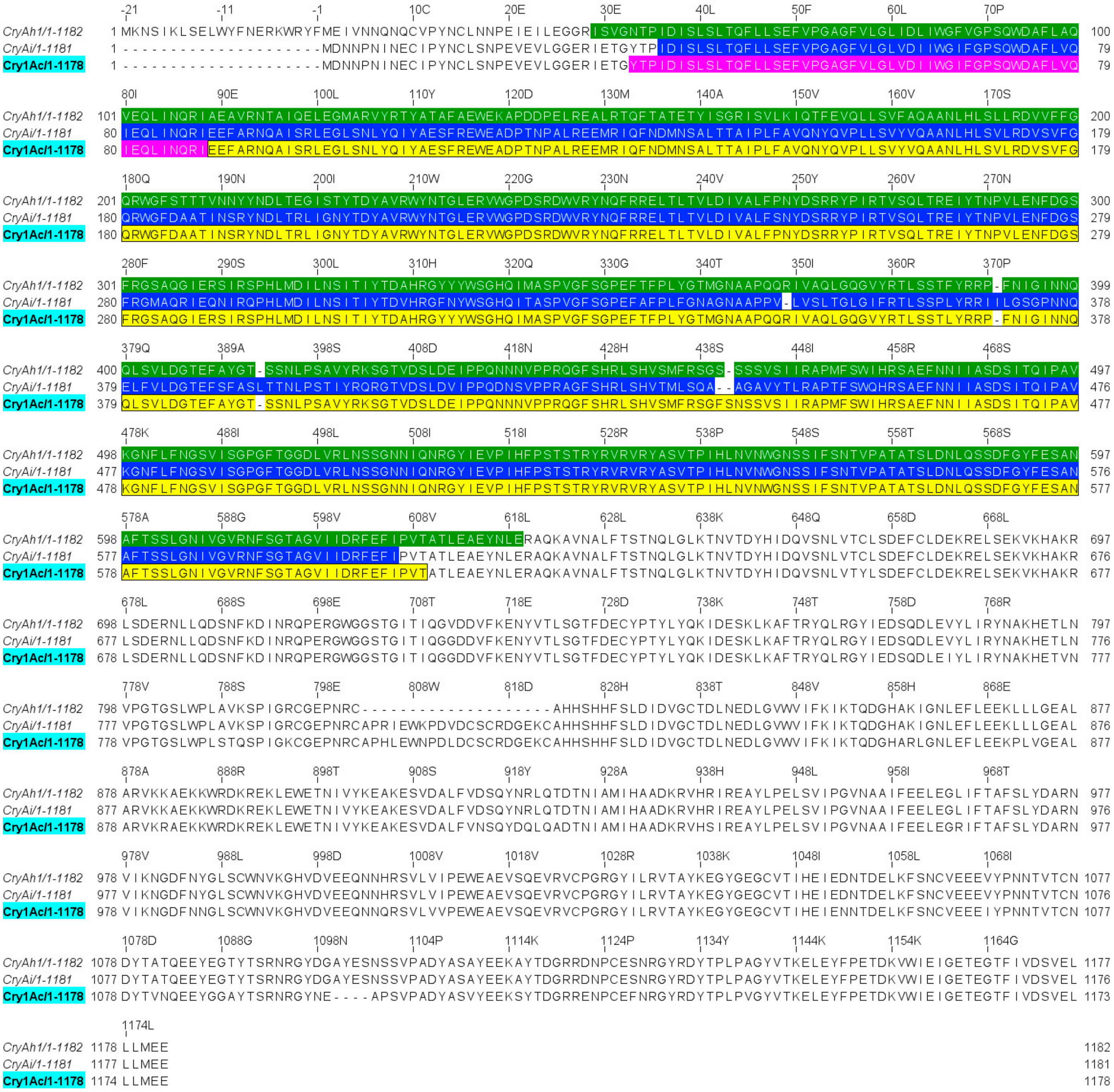

Figure S5. Sequence alignment of Cry1A proteins. The alignment was performed using Clustal Omega and visualized by Jalview using Cry1Ac sequence was used as the reference. The active form of Cry1Ai (residues 36–605) was highlighted in blue and Cry1Ah (residues 50–639) was highlighted in green. The Cry1Ac N-terminal extension (residues before its five-helix bundle) was indicated as magenta, while its three-domain core is colored in yellow.

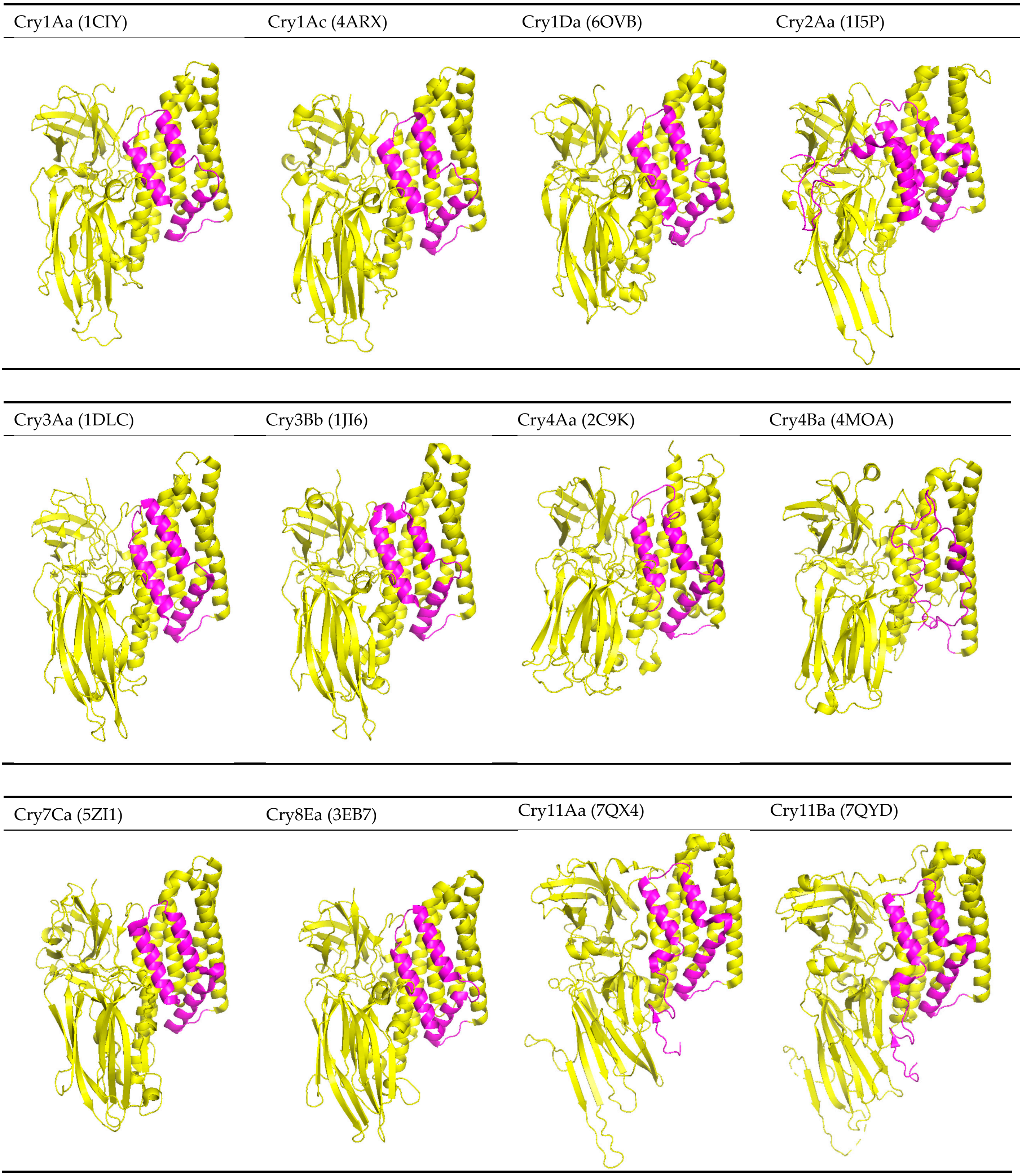

Figure S6. Structures of three-domain Cry proteins possess with the extra amino acid beyond five-helix bundle. The N-terminal region is highlighted in magenta while the three-domain core start with five-helix colored in yellow. PDB ID of each structure shown in bracket.

Table S1. Primers used for making Cry5B constructs.

Cry5B 1-EcoRI-F:	5′-GGTCGGGATCCGAATTCCATGGCAACAATTAATGAGTTGTATC-3′
Cry5B 112-EcoRI-F:	5′-GGTCGGGATCCGAATTCCATGAAAGATCAACAGTTGTTTAATG-3′
Cry5B 12-EcoRI-F	5′-GGTCGGGATCCGAATTCCTATAATGTGCTAGCTCATCCAATTAAA-3′
Cry5B 21-EcoRI-F	5′-GGTCGGGATCCGAATTCCGAAGTCGATGATCCTTATTC-3′
Cry5B 27-EcoRI-F	5′-GGTCGGGATCCGAATTCCTCTTGGTCAAAT TTATTAAAGG-3′
Cry5B 698-XholI-R:	5′-GTGGTGGTGGTGGTGGTGCTCGAGTTATTGGATTTTTGGAACAAACTCA-3′
Cry5B 772-XholI-R:	5′-TGGTGGTGGTGGTGGTGCTCGAGTTATGCAACTAATGCATTTACTTGT-3′
Y495A-a	5′-TTTGAGGAGGACGTAAAGCATTAATCCATGCATCACAAATGCATGAACC-3′
Y495A-b	5′-GGTTCATGCATTTGTGATGCATGGATTAATGCTTTACGTCCTCCTCAAA-3′
Y495F-a	5′-TTGAGGAGGACGTAAAAAATTAATCCATGCATCACAAATGCATGA-3′
Y495F-b	5′-TCATGCATTTGTGATGCATGGATTAATTTTTTACGTCCTCCTCAA-3′
Y495W-a	5′-GTTTGAGGAGGACGTAACCAATTAATCCATGCATCACAAATGCATGAACC-3′
Y495W-b	5′-GGTTCATGCATTTGTGATGCATGGATTAATTGGTTACGTCCTCCTCAAAC-3′
